# Ethyl 1-*sec*-butyl-2-*p*-tolyl-1*H*-benzimidazole-5-carboxyl­ate

**DOI:** 10.1107/S1600536810015242

**Published:** 2010-04-30

**Authors:** Natarajan Arumugam, Aisyah Saad Abdul Rahim, Hasnah Osman, Chin Sing Yeap, Hoong-Kun Fun

**Affiliations:** a School of Pharmaceutical Sciences, Universiti Sains Malaysia, 11800 USM, Penang, Malaysia; b School of Chemical Sciences, Universiti Sains Malaysia, 11800 USM, Penang, Malaysia; c X-ray Crystallography Unit, School of Physics, Universiti Sains Malaysia, 11800 USM, Penang, Malaysia

## Abstract

In the title compound, C_21_H_24_N_2_O_2_, the butyl group is disordered over two orientations with refined site occupancies of 0.883 (3) and 0.117 (3). The dihedral angle between the mean plane of benzimidazole ring system and the benzene ring is 39.32 (4)° and the dihedral angle between the mean plane of carboxyl­ate group and the benzimidazole ring system is 6.87 (5)°. A weak intra­molecular C—H⋯π inter­action may have some influence on the conformation of the mol­ecule. In the crystal structure, mol­ecules are linked into infinite chains along the *b* axis by weak inter­molecular C—H⋯O hydrogen bonds.

## Related literature

For background information on benzimidazole derivatives, their biological activity and medical applications, see: Richter (1997 ▶); Can-Eke *et al.* (1998 ▶); Evans *et al.* (1997 ▶); Garuti *et al.* (2000 ▶); Sondhi *et al.* (2005 ▶). For the synthesis of the title compound and related structures, see: Arumugam *et al.* (2010*a* ▶,*b* ▶,*c* ▶). For the stability of the temperature controller used for the data collection, see: Cosier & Glazer (1986 ▶).
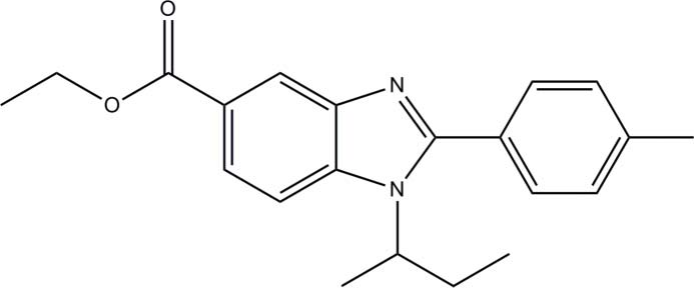


## Experimental

### 

#### Crystal data

C_21_H_24_N_2_O_2_
*M* *_r_* = 336.42
Monoclinic, 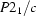

*a* = 10.6093 (7) Å
*b* = 12.5617 (9) Å
*c* = 13.6025 (10) Å
β = 96.412 (2)°
*V* = 1801.5 (2) Å^3^
*Z* = 4
Mo *K*α radiation
μ = 0.08 mm^−1^
*T* = 100 K
0.46 × 0.29 × 0.24 mm

#### Data collection

Bruker APEXII DUO CCD area-detector diffractometer
Absorption correction: multi-scan (*SADABS*; Bruker, 2009 ▶) *T*_min_ = 0.964, *T*_max_ = 0.981
31247 measured reflections
8425 independent reflections
6598 reflections with *I* > 2σ(*I*)
*R*_int_ = 0.050

#### Refinement

*R*[*F* ^2^ > 2σ(*F* ^2^)] = 0.051
*wR*(*F* ^2^) = 0.177
*S* = 1.08
8425 reflections
249 parameters
H-atom parameters constrained
Δρ_max_ = 0.58 e Å^−3^
Δρ_min_ = −0.35 e Å^−3^

### 

Data collection: *APEX2* (Bruker, 2009 ▶); cell refinement: *SAINT* (Bruker, 2009 ▶); data reduction: *SAINT*; program(s) used to solve structure: *SHELXTL* (Sheldrick, 2008 ▶); program(s) used to refine structure: *SHELXTL*; molecular graphics: *SHELXTL*; software used to prepare material for publication: *SHELXTL* and *PLATON* (Spek, 2009 ▶).

## Supplementary Material

Crystal structure: contains datablocks global, I. DOI: 10.1107/S1600536810015242/lh5024sup1.cif

Structure factors: contains datablocks I. DOI: 10.1107/S1600536810015242/lh5024Isup2.hkl

Additional supplementary materials:  crystallographic information; 3D view; checkCIF report

## Figures and Tables

**Table 1:** Hydrogen-bond geometry (Å, °) *Cg*1 is centroid of the N1/C7/N2/C13/C8 ring.

*D*—H⋯*A*	*D*—H	H⋯*A*	*D*⋯*A*	*D*—H⋯*A*
C12—H12*A*⋯O1^i^	0.93	2.58	3.5007 (13)	173
C20—H20*C*⋯*Cg*1	0.96	2.72	3.3432 (13)	123

Symmetry code: (i) 
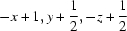
.

**Fig. 1.**
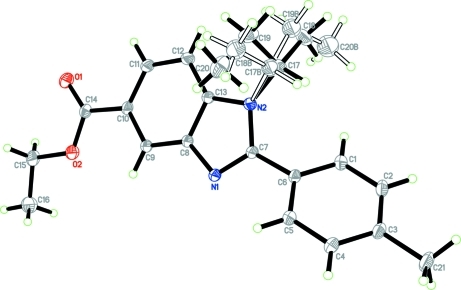
The molecular structure of the title compound with atom labels and 50% probability displacement ellipsoids for non-H atoms. All disorder components are shown.

**Fig. 2.**
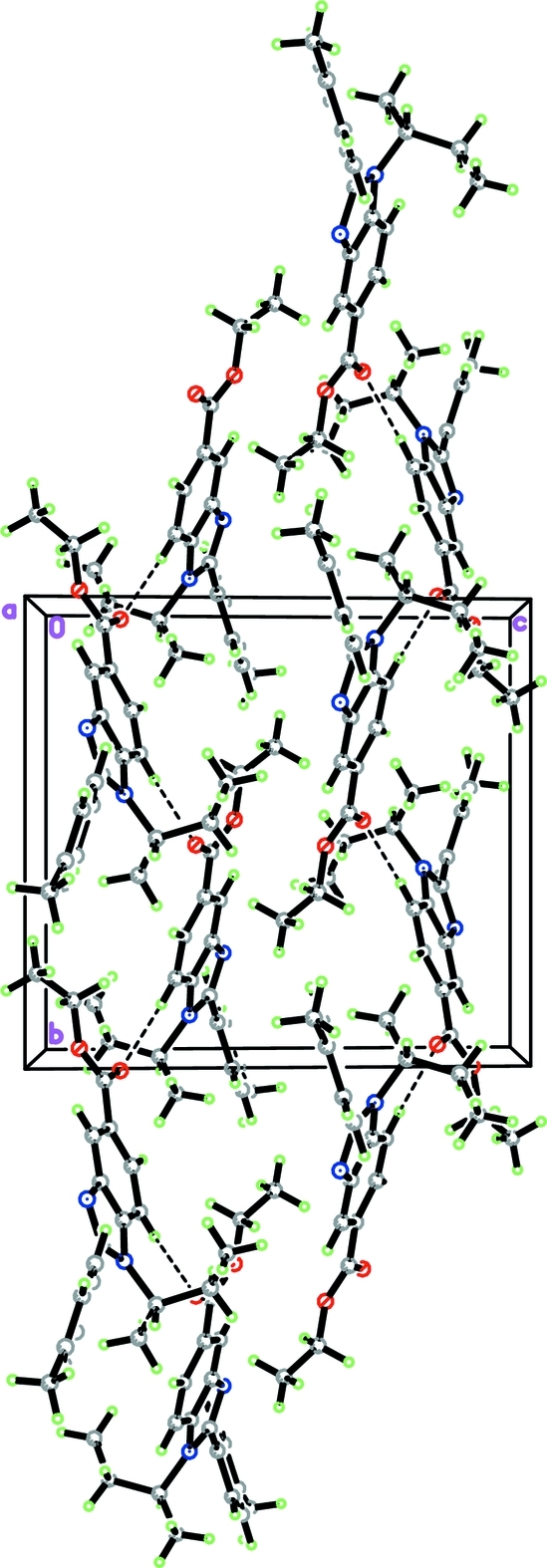
The crystal packing of the title compound, viewed along the a axis, showing one-dimensional chains along the b axis. Intermolecular hydrogen bonds are shown as dashed lines. Only the major disorder component is shown.

## supplementary crystallographic information

### Comment

Benzimidazoles are important heterocyclic compounds from the view point of their biological activities. Substituted benzimidazole derivatives have diverse therapeutic applications as they exhibit antiulcerative (Richter, 1997), antioxidant (Can-Eke *et al.*, 1998), anti-HIV-1 (Evans *et al.*, 1997), antiproliferative (Garuti *et al.*, 2000) and antikinase (Sondhi *et al.*, 2005) activities. In view of their importance, the crystal structure determination of the title compound was carried out and the results are presented herein.

The geometric parameters of the title compound (Fig. 1) are comparable to those closely related structures (Arumugam *et al.*, 2010a,b,c). The butyl group is disordered over two positions with refined site-occupancies of 0.883 (3) and 0.117 (3). The dihedral angle between the mean plane of benzimidazole ring system (C7/N1/C8–C13/N2) and the benzene ring (C1–C6) is 39.32 (4)°. The mean plane of carboxylate group (O1/O2/C14–C16) is slightly twisted from the mean plane of benzimidazole ring system with a dihedral angle of 6.87 (5)°. In the crystal structure, the molecules are linked into infinite one-dimensional chains along *b* axis by intermolecular C12—H12A···O1^i^ hydrogen bonds (Fig. 2, Table 1). A weak intramolecular C20—H20C···*Cg*1 interaction may have some influence on the conformation of the molecule (Table 1).

### Experimental

The title compound was synthesised using the previous procedures (Arumugam *et al.*, 2010a,b,c) and recrystallized from EtOAc by slow evaporation technique.

### Refinement

All H atoms were positioned geometrically and refined using a riding model, with C–H = 0.93–0.98 Å and *U*_iso_(H) = 1.2 or 1.5 *U*_eq_(C). The rotating group model was applied for the methyl groups. The minor disorder component is refined isotropically.

### Crystal data

**Table:**

C_21_H_24_N_2_O_2_	*F*(000) = 720
*M**_r_* = 336.42	*D*_x_ = 1.240 Mg m^−^^3^
Monoclinic, *P*2_1_/*c*	Mo *K*α radiation, λ = 0.71073 Å
Hall symbol: -P 2ybc	Cell parameters from 9348 reflections
*a* = 10.6093 (7) Å	θ = 2.5–35.7°
*b* = 12.5617 (9) Å	µ = 0.08 mm^−^^1^
*c* = 13.6025 (10) Å	*T* = 100 K
β = 96.412 (2)°	Block, colourless
*V* = 1801.5 (2) Å^3^	0.46 × 0.29 × 0.24 mm
*Z* = 4	

### Data collection

**Table:**

Bruker APEXII DUO CCD area-detector diffractometer	8425 independent reflections
Radiation source: fine-focus sealed tube	6598 reflections with *I* > 2σ(*I*)
graphite	*R*_int_ = 0.050
φ and ω scans	θ_max_ = 35.9°, θ_min_ = 1.9°
Absorption correction: multi-scan (*SADABS*; Bruker, 2009)	*h* = −17→17
*T*_min_ = 0.964, *T*_max_ = 0.981	*k* = −20→18
31247 measured reflections	*l* = −22→22

### Refinement

**Table:**

Refinement on *F*^2^	Primary atom site location: structure-invariant direct methods
Least-squares matrix: full	Secondary atom site location: difference Fourier map
*R*[*F*^2^ > 2σ(*F*^2^)] = 0.051	Hydrogen site location: inferred from neighbouring sites
*wR*(*F*^2^) = 0.177	H-atom parameters constrained
*S* = 1.08	*w* = 1/[σ^2^(*F*_o_^2^) + (0.0965*P*)^2^ + 0.2974*P*] where *P* = (*F*_o_^2^ + 2*F*_c_^2^)/3
8425 reflections	(Δ/σ)_max_ = 0.001
249 parameters	Δρ_max_ = 0.58 e Å^−^^3^
0 restraints	Δρ_min_ = −0.35 e Å^−^^3^

### Special details

**Table:**

Experimental. The crystal was placed in the cold stream of an Oxford Cryosystems Cobra open-flow nitrogen cryostat (Cosier & Glazer, 1986) operating at 100.0 (1) K.
Geometry. All esds (except the esd in the dihedral angle between two l.s. planes) are estimated using the full covariance matrix. The cell esds are taken into account individually in the estimation of esds in distances, angles and torsion angles; correlations between esds in cell parameters are only used when they are defined by crystal symmetry. An approximate (isotropic) treatment of cell esds is used for estimating esds involving l.s. planes.
Refinement. Refinement of F^2^ against ALL reflections. The weighted R-factor wR and goodness of fit S are based on F^2^, conventional R-factors R are based on F, with F set to zero for negative F^2^. The threshold expression of F^2^ > 2sigma(F^2^) is used only for calculating R-factors(gt) etc. and is not relevant to the choice of reflections for refinement. R-factors based on F^2^ are statistically about twice as large as those based on F, and R- factors based on ALL data will be even larger.

### Fractional atomic coordinates and isotropic or equivalent isotropic displacement parameters (Å^2^)

**Table:**

	*x*	*y*	*z*	*U*_iso_*/*U*_eq_	Occ. (<1)
O1	0.49342 (7)	0.02746 (7)	0.17217 (6)	0.02380 (16)	
O2	0.65726 (7)	−0.02944 (6)	0.09409 (6)	0.02268 (16)	
N1	0.97240 (7)	0.27803 (6)	0.12066 (6)	0.01543 (14)	
N2	0.88175 (7)	0.41780 (6)	0.19145 (6)	0.01655 (14)	
C1	1.08554 (9)	0.54848 (8)	0.10257 (7)	0.01777 (16)	
H1A	1.0063	0.5810	0.0955	0.021*	
C2	1.19164 (9)	0.60526 (8)	0.08166 (7)	0.01913 (17)	
H2A	1.1826	0.6757	0.0611	0.023*	
C3	1.31155 (9)	0.55829 (8)	0.09096 (7)	0.01821 (16)	
C4	1.32288 (9)	0.45317 (8)	0.12279 (8)	0.02007 (17)	
H4A	1.4023	0.4209	0.1299	0.024*	
C5	1.21734 (8)	0.39545 (8)	0.14411 (7)	0.01823 (16)	
H5A	1.2267	0.3251	0.1651	0.022*	
C6	1.09738 (8)	0.44266 (7)	0.13421 (7)	0.01533 (15)	
C7	0.98590 (8)	0.37775 (7)	0.15004 (7)	0.01501 (15)	
C8	0.85266 (8)	0.24973 (7)	0.14314 (6)	0.01444 (14)	
C9	0.78890 (8)	0.15330 (7)	0.12648 (7)	0.01558 (15)	
H9A	0.8262	0.0964	0.0969	0.019*	
C10	0.66749 (8)	0.14490 (7)	0.15547 (7)	0.01544 (15)	
C11	0.61067 (8)	0.23144 (8)	0.19943 (7)	0.01795 (16)	
H11A	0.5293	0.2235	0.2176	0.022*	
C12	0.67222 (9)	0.32803 (8)	0.21644 (7)	0.01830 (16)	
H12A	0.6344	0.3848	0.2457	0.022*	
C13	0.79460 (8)	0.33574 (7)	0.18720 (7)	0.01546 (15)	
C14	0.59579 (9)	0.04345 (8)	0.14279 (7)	0.01746 (16)	
C15	0.59689 (11)	−0.13214 (8)	0.07980 (9)	0.0248 (2)	
H15A	0.5092	−0.1239	0.0519	0.030*	
H15B	0.5987	−0.1694	0.1424	0.030*	
C16	0.67033 (12)	−0.19304 (10)	0.00983 (10)	0.0309 (2)	
H16A	0.6344	−0.2628	−0.0007	0.046*	
H16B	0.7572	−0.1992	0.0377	0.046*	
H16C	0.6662	−0.1560	−0.0522	0.046*	
C17	0.88130 (11)	0.51250 (9)	0.25531 (10)	0.0177 (2)	0.883 (3)
H17A	0.9632	0.5481	0.2535	0.021*	0.883 (3)
C18	0.77857 (12)	0.59232 (10)	0.21723 (12)	0.0295 (3)	0.883 (3)
H18A	0.7830	0.6054	0.1482	0.044*	0.883 (3)
H18B	0.7915	0.6578	0.2533	0.044*	0.883 (3)
H18C	0.6967	0.5639	0.2263	0.044*	0.883 (3)
C19	0.87392 (12)	0.47809 (11)	0.36205 (9)	0.0252 (3)	0.883 (3)
H19A	0.8852	0.5399	0.4048	0.030*	0.883 (3)
H19B	0.7904	0.4489	0.3678	0.030*	0.883 (3)
C20	0.97390 (13)	0.39537 (13)	0.39635 (9)	0.0283 (3)	0.883 (3)
H20A	0.9696	0.3794	0.4649	0.042*	0.883 (3)
H20B	1.0565	0.4228	0.3880	0.042*	0.883 (3)
H20C	0.9588	0.3317	0.3578	0.042*	0.883 (3)
C17B	0.9123 (10)	0.4910 (8)	0.2883 (8)	0.0205 (17)*	0.117 (3)
H17B	0.9914	0.5291	0.2810	0.025*	0.117 (3)
C18B	0.9279 (12)	0.4407 (10)	0.3838 (8)	0.028 (2)*	0.117 (3)
H18D	0.9359	0.4942	0.4345	0.042*	0.117 (3)
H18E	1.0028	0.3973	0.3895	0.042*	0.117 (3)
H18F	0.8553	0.3970	0.3912	0.042*	0.117 (3)
C19B	0.8068 (10)	0.5725 (9)	0.2776 (8)	0.028 (2)*	0.117 (3)
H19C	0.8237	0.6249	0.3298	0.034*	0.117 (3)
H19D	0.7281	0.5370	0.2878	0.034*	0.117 (3)
C20B	0.7858 (14)	0.6360 (12)	0.1703 (11)	0.041 (3)*	0.117 (3)
H20D	0.7288	0.6946	0.1751	0.061*	0.117 (3)
H20E	0.7507	0.5879	0.1196	0.061*	0.117 (3)
H20F	0.8659	0.6623	0.1541	0.061*	0.117 (3)
C21	1.42509 (11)	0.62003 (10)	0.06554 (8)	0.0258 (2)	
H21A	1.5001	0.5936	0.1038	0.039*	
H21B	1.4142	0.6940	0.0802	0.039*	
H21C	1.4334	0.6119	−0.0036	0.039*	

### Atomic displacement parameters (Å^2^)

**Table:**

	*U*^11^	*U*^22^	*U*^33^	*U*^12^	*U*^13^	*U*^23^
O1	0.0180 (3)	0.0222 (4)	0.0325 (4)	−0.0070 (2)	0.0084 (3)	−0.0006 (3)
O2	0.0226 (3)	0.0155 (3)	0.0316 (4)	−0.0089 (2)	0.0100 (3)	−0.0062 (3)
N1	0.0142 (3)	0.0130 (3)	0.0200 (3)	−0.0026 (2)	0.0059 (2)	−0.0017 (2)
N2	0.0157 (3)	0.0132 (3)	0.0217 (3)	−0.0031 (2)	0.0066 (3)	−0.0045 (2)
C1	0.0181 (4)	0.0147 (4)	0.0207 (4)	−0.0026 (3)	0.0031 (3)	0.0002 (3)
C2	0.0231 (4)	0.0151 (4)	0.0195 (4)	−0.0057 (3)	0.0035 (3)	0.0004 (3)
C3	0.0187 (4)	0.0190 (4)	0.0175 (4)	−0.0073 (3)	0.0046 (3)	−0.0017 (3)
C4	0.0163 (4)	0.0195 (4)	0.0252 (4)	−0.0035 (3)	0.0057 (3)	0.0000 (3)
C5	0.0160 (3)	0.0152 (4)	0.0242 (4)	−0.0019 (3)	0.0056 (3)	0.0004 (3)
C6	0.0153 (3)	0.0138 (4)	0.0174 (3)	−0.0034 (3)	0.0044 (3)	−0.0016 (3)
C7	0.0141 (3)	0.0135 (4)	0.0181 (3)	−0.0025 (2)	0.0047 (3)	−0.0014 (3)
C8	0.0139 (3)	0.0123 (3)	0.0178 (3)	−0.0025 (2)	0.0045 (3)	−0.0015 (3)
C9	0.0155 (3)	0.0127 (3)	0.0192 (4)	−0.0028 (3)	0.0048 (3)	−0.0018 (3)
C10	0.0153 (3)	0.0139 (4)	0.0175 (3)	−0.0037 (3)	0.0037 (3)	−0.0001 (3)
C11	0.0145 (3)	0.0184 (4)	0.0217 (4)	−0.0024 (3)	0.0056 (3)	−0.0012 (3)
C12	0.0153 (3)	0.0168 (4)	0.0238 (4)	−0.0016 (3)	0.0068 (3)	−0.0038 (3)
C13	0.0142 (3)	0.0135 (4)	0.0193 (4)	−0.0026 (3)	0.0047 (3)	−0.0028 (3)
C14	0.0166 (3)	0.0162 (4)	0.0197 (4)	−0.0044 (3)	0.0027 (3)	0.0008 (3)
C15	0.0265 (5)	0.0175 (4)	0.0314 (5)	−0.0108 (3)	0.0076 (4)	−0.0043 (4)
C16	0.0303 (5)	0.0212 (5)	0.0426 (6)	−0.0081 (4)	0.0110 (5)	−0.0081 (4)
C17	0.0177 (4)	0.0137 (4)	0.0224 (5)	−0.0019 (3)	0.0046 (4)	−0.0060 (4)
C18	0.0230 (5)	0.0171 (5)	0.0492 (9)	0.0041 (4)	0.0076 (5)	−0.0052 (5)
C19	0.0232 (5)	0.0321 (7)	0.0213 (5)	−0.0070 (5)	0.0070 (4)	−0.0101 (4)
C20	0.0289 (6)	0.0361 (7)	0.0195 (5)	−0.0105 (5)	0.0007 (4)	0.0019 (4)
C21	0.0246 (4)	0.0261 (5)	0.0280 (5)	−0.0124 (4)	0.0090 (4)	−0.0016 (4)

### Geometric parameters (Å, °)

**Table:**

O1—C14	1.2151 (11)	C15—H15A	0.9700
O2—C14	1.3412 (12)	C15—H15B	0.9700
O2—C15	1.4438 (12)	C16—H16A	0.9600
N1—C7	1.3178 (12)	C16—H16B	0.9600
N1—C8	1.3858 (11)	C16—H16C	0.9600
N2—C13	1.3817 (11)	C17—C19	1.5253 (18)
N2—C7	1.3895 (11)	C17—C18	1.5281 (19)
N2—C17	1.4733 (13)	C17—H17A	0.9800
N2—C17B	1.609 (10)	C18—H18A	0.9600
C1—C2	1.3886 (13)	C18—H18B	0.9600
C1—C6	1.3985 (13)	C18—H18C	0.9600
C1—H1A	0.9300	C19—C20	1.521 (2)
C2—C3	1.3952 (14)	C19—H19A	0.9700
C2—H2A	0.9300	C19—H19B	0.9700
C3—C4	1.3907 (14)	C20—H20A	0.9600
C3—C21	1.5051 (13)	C20—H20B	0.9600
C4—C5	1.3914 (13)	C20—H20C	0.9600
C4—H4A	0.9300	C17B—C18B	1.438 (16)
C5—C6	1.3969 (13)	C17B—C19B	1.512 (15)
C5—H5A	0.9300	C17B—H17B	0.9800
C6—C7	1.4720 (12)	C18B—H18D	0.9600
C8—C9	1.3935 (12)	C18B—H18E	0.9600
C8—C13	1.4101 (12)	C18B—H18F	0.9600
C9—C10	1.3926 (12)	C19B—C20B	1.656 (19)
C9—H9A	0.9300	C19B—H19C	0.9700
C10—C11	1.4083 (13)	C19B—H19D	0.9700
C10—C14	1.4842 (13)	C20B—H20D	0.9600
C11—C12	1.3851 (13)	C20B—H20E	0.9600
C11—H11A	0.9300	C20B—H20F	0.9600
C12—C13	1.4031 (12)	C21—H21A	0.9600
C12—H12A	0.9300	C21—H21B	0.9600
C15—C16	1.5047 (17)	C21—H21C	0.9600
			
C14—O2—C15	116.33 (8)	O2—C15—H15B	110.4
C7—N1—C8	104.38 (7)	C16—C15—H15B	110.4
C13—N2—C7	105.76 (7)	H15A—C15—H15B	108.6
C13—N2—C17	125.52 (8)	C15—C16—H16A	109.5
C7—N2—C17	125.91 (8)	C15—C16—H16B	109.5
C13—N2—C17B	122.2 (4)	H16A—C16—H16B	109.5
C7—N2—C17B	116.2 (4)	C15—C16—H16C	109.5
C2—C1—C6	120.28 (9)	H16A—C16—H16C	109.5
C2—C1—H1A	119.9	H16B—C16—H16C	109.5
C6—C1—H1A	119.9	N2—C17—C19	109.65 (10)
C1—C2—C3	121.07 (9)	N2—C17—C18	112.27 (11)
C1—C2—H2A	119.5	C19—C17—C18	113.46 (10)
C3—C2—H2A	119.5	N2—C17—H17A	107.0
C4—C3—C2	118.42 (8)	C19—C17—H17A	107.0
C4—C3—C21	121.15 (9)	C18—C17—H17A	107.0
C2—C3—C21	120.42 (10)	C20—C19—C17	112.18 (10)
C3—C4—C5	121.07 (9)	C20—C19—H19A	109.2
C3—C4—H4A	119.5	C17—C19—H19A	109.2
C5—C4—H4A	119.5	C20—C19—H19B	109.2
C4—C5—C6	120.29 (9)	C17—C19—H19B	109.2
C4—C5—H5A	119.9	H19A—C19—H19B	107.9
C6—C5—H5A	119.9	C18B—C17B—C19B	113.2 (9)
C5—C6—C1	118.86 (8)	C18B—C17B—N2	118.7 (8)
C5—C6—C7	119.27 (8)	C19B—C17B—N2	103.2 (8)
C1—C6—C7	121.71 (8)	C18B—C17B—H17B	107.0
N1—C7—N2	113.75 (7)	C19B—C17B—H17B	107.0
N1—C7—C6	122.96 (8)	N2—C17B—H17B	107.0
N2—C7—C6	123.17 (8)	C17B—C18B—H18D	109.5
N1—C8—C9	128.86 (8)	C17B—C18B—H18E	109.5
N1—C8—C13	110.60 (7)	H18D—C18B—H18E	109.5
C9—C8—C13	120.53 (8)	C17B—C18B—H18F	109.5
C10—C9—C8	117.86 (8)	H18D—C18B—H18F	109.5
C10—C9—H9A	121.1	H18E—C18B—H18F	109.5
C8—C9—H9A	121.1	C17B—C19B—C20B	115.8 (9)
C9—C10—C11	121.00 (8)	C17B—C19B—H19C	108.3
C9—C10—C14	120.69 (8)	C20B—C19B—H19C	108.3
C11—C10—C14	118.29 (8)	C17B—C19B—H19D	108.3
C12—C11—C10	122.07 (8)	C20B—C19B—H19D	108.3
C12—C11—H11A	119.0	H19C—C19B—H19D	107.4
C10—C11—H11A	119.0	C19B—C20B—H20D	109.5
C11—C12—C13	116.55 (8)	C19B—C20B—H20E	109.5
C11—C12—H12A	121.7	H20D—C20B—H20E	109.5
C13—C12—H12A	121.7	C19B—C20B—H20F	109.5
N2—C13—C12	132.51 (8)	H20D—C20B—H20F	109.5
N2—C13—C8	105.51 (7)	H20E—C20B—H20F	109.5
C12—C13—C8	121.98 (8)	C3—C21—H21A	109.5
O1—C14—O2	123.46 (9)	C3—C21—H21B	109.5
O1—C14—C10	124.68 (9)	H21A—C21—H21B	109.5
O2—C14—C10	111.86 (8)	C3—C21—H21C	109.5
O2—C15—C16	106.63 (8)	H21A—C21—H21C	109.5
O2—C15—H15A	110.4	H21B—C21—H21C	109.5
C16—C15—H15A	110.4		
			
C6—C1—C2—C3	0.45 (15)	C17B—N2—C13—C12	44.5 (5)
C1—C2—C3—C4	−0.67 (14)	C7—N2—C13—C8	0.23 (10)
C1—C2—C3—C21	178.44 (9)	C17—N2—C13—C8	−161.60 (10)
C2—C3—C4—C5	0.58 (15)	C17B—N2—C13—C8	−135.9 (5)
C21—C3—C4—C5	−178.53 (9)	C11—C12—C13—N2	179.26 (10)
C3—C4—C5—C6	−0.26 (15)	C11—C12—C13—C8	−0.27 (14)
C4—C5—C6—C1	0.02 (14)	N1—C8—C13—N2	−0.09 (10)
C4—C5—C6—C7	175.50 (9)	C9—C8—C13—N2	−179.20 (8)
C2—C1—C6—C5	−0.12 (14)	N1—C8—C13—C12	179.55 (9)
C2—C1—C6—C7	−175.48 (9)	C9—C8—C13—C12	0.43 (14)
C8—N1—C7—N2	0.25 (10)	C15—O2—C14—O1	−1.73 (15)
C8—N1—C7—C6	−175.82 (8)	C15—O2—C14—C10	178.60 (8)
C13—N2—C7—N1	−0.31 (11)	C9—C10—C14—O1	174.76 (10)
C17—N2—C7—N1	161.42 (10)	C11—C10—C14—O1	−3.91 (15)
C17B—N2—C7—N1	138.9 (4)	C9—C10—C14—O2	−5.57 (13)
C13—N2—C7—C6	175.74 (9)	C11—C10—C14—O2	175.76 (8)
C17—N2—C7—C6	−22.52 (15)	C14—O2—C15—C16	170.62 (10)
C17B—N2—C7—C6	−45.1 (5)	C13—N2—C17—C19	51.97 (14)
C5—C6—C7—N1	−38.44 (13)	C7—N2—C17—C19	−106.28 (11)
C1—C6—C7—N1	136.90 (10)	C17B—N2—C17—C19	−36.7 (9)
C5—C6—C7—N2	145.86 (9)	C13—N2—C17—C18	−75.12 (14)
C1—C6—C7—N2	−38.79 (13)	C7—N2—C17—C18	126.63 (11)
C7—N1—C8—C9	178.93 (9)	C17B—N2—C17—C18	−163.7 (10)
C7—N1—C8—C13	−0.10 (10)	N2—C17—C19—C20	51.24 (13)
N1—C8—C9—C10	−179.49 (9)	C18—C17—C19—C20	177.66 (10)
C13—C8—C9—C10	−0.55 (13)	C13—N2—C17B—C18B	43.5 (10)
C8—C9—C10—C11	0.54 (14)	C7—N2—C17B—C18B	−88.4 (9)
C8—C9—C10—C14	−178.09 (8)	C17—N2—C17B—C18B	149.4 (15)
C9—C10—C11—C12	−0.41 (15)	C13—N2—C17B—C19B	−82.6 (7)
C14—C10—C11—C12	178.26 (9)	C7—N2—C17B—C19B	145.4 (5)
C10—C11—C12—C13	0.26 (14)	C17—N2—C17B—C19B	23.2 (7)
C7—N2—C13—C12	−179.36 (10)	C18B—C17B—C19B—C20B	178.5 (9)
C17—N2—C13—C12	18.81 (17)	N2—C17B—C19B—C20B	−51.9 (10)

### Hydrogen-bond geometry (Å, °)

**Table:**

Cg1 is centroid of the N1/C7/N2/C13/C8 ring.

**Table:**

*D*—H···*A*	*D*—H	H···*A*	*D*···*A*	*D*—H···*A*
C12—H12A···O1^i^	0.93	2.58	3.5007 (13)	173
C20—H20C···Cg1	0.96	2.72	3.3432 (13)	123

Symmetry codes: (i) −*x*+1, *y*+1/2, −*z*+1/2.
